# Systematic review and meta-analysis of the duration of clinical effect of onabotulinumtoxinA in cervical dystonia

**DOI:** 10.1186/1471-2377-14-91

**Published:** 2014-04-27

**Authors:** Wallace A Marsh, Deirdre M Monroe, Mitchell F Brin, Conor J Gallagher

**Affiliations:** 1 Shenandoah University, Winchester, VA, USA; 2 Allergan, Inc., Irvine, CA, USA; 3 University of California Irvine, Irvine, CA, USA

**Keywords:** Cervical dystonia, Duration of effect, OnabotulinumtoxinA

## Abstract

**Background:**

Botulinum toxins are considered first-line therapy for treatment of cervical dystonia (CD) and must be injected on a repeat basis. Understanding the duration of clinical benefit of botulinum toxins and its impact on health care utilization are thus important in the contemporary environment. However, there is currently no overall consensus on the duration of effect of onabotulinumtoxinA in the treatment of CD. We performed a systematic review and meta-analysis to identify the duration of effect of onabotulinumtoxinA in CD and investigate factors that may influence it.

**Methods:**

A systematic literature search identified prospective or retrospective studies reporting duration of effect of onabotulinumtoxinA for the treatment of CD. Inclusion criteria included peer-reviewed, non-review, English-language articles published between January 1980 and January 2013. A formal meta-analysis using Comprehensive Meta-Analysis Version 2 was conducted to identify the duration of effect of onabotulinumtoxinA in the treatment of CD; both fixed and random effects models were performed. Subgroup analyses were performed to identify factors that influenced the duration of effect of onabotulinumtoxinA.

**Results:**

A total of 18 studies (including >1,900 patients) met the inclusion criteria and were used for the meta-analysis. The mean duration of effect of onabotulinumtoxinA in CD was found to be 93.2 days (95% CI 91.8-94.6 days) for the fixed effects model and 95.2 days (95% CI 88.9-101.4 days) for the random effects model. A meta-regression found that dose of onabotulinumtoxinA and country of origin influenced the duration of effect of onabotulinumtoxinA, whereas quality score of the article and study type did not. In particular, doses ≥180 Units were associated with longer durations of effect than doses <180 Units (107-109 days vs. 86-88 days, respectively; p < 0.01). Limitations included pooling studies that used discrete definitions of duration and had different designs and study quality.

**Conclusions:**

Based on the published literature, the mean duration of effect of onabotulinumtoxinA in CD was 93-95 days (13.2-13.5 weeks). This suggests that, in general, patients with CD treated with onabotulinumtoxinA should require ~4 treatments per year.

## Background

Cervical dystonia (CD) is a disabling, painful condition involving involuntary movement and posturing of the head and neck [1]. Botulinum toxin injections have been the standard of care in the symptomatic management of this condition since their approval for this indication in the early 2000s. When injected into skeletal muscle, botulinum toxins act to disrupt neurotransmitter release at the neuromuscular junction, inducing a transient weakening of the muscle. Because of the temporary nature of the effect of botulinum toxins, they need to be readministered regularly to maintain clinical improvement [2,3].

The duration of the clinical benefit produced by a botulinum toxin is thus an important factor in treatment. A longer duration of effect will reduce the frequency of reinjection, benefiting patients in terms of decreasing the inconvenience of undergoing the injection treatment and additional co-pay costs, benefiting physicians in terms of patient scheduling and optimizing wait times for appointments, and benefiting the health care system in general in terms of reduced heath care utilization costs.

The duration of effect of a botulinum toxin can likely be influenced by multiple factors. In particular, the dose of a botulinum toxin may affect its duration, with higher doses generally accepted as producing a longer clinical benefit, up to a certain point. As the various commercially available botulinum toxin products are not interchangeable and differ from each other in their units of biologic activity, it is likely that differences may exist in duration of clinical benefit even if they are used at their approved unit doses.

Since 2000, onabotulinumtoxinA (BOTOX®; Allergan, Inc.) has been approved in the US to reduce the severity of abnormal head position and neck pain in adult patients with CD [4]. The typical inter-injection interval employed in clinical practice is 3-4 months [5], with the label suggesting a minimum inter-injection interval of 3 months [4]. Although some individual studies have reported the duration of effect of onabotulinumtoxinA in the treatment of CD [3,6-8], no overall consensus on duration of effect currently exists, and no prospective trials have been performed specifically to assess duration. The trial that formed the basis of the Food and Drug Administration approval of onabotulinumtoxinA for the treatment of CD was limited by the study length and was unable to fully assess duration of benefit [9]. Furthermore, no dose-ranging studies have been performed with onabotulinumtoxinA, so data on whether there is a dose-response relationship for the duration of effect of onabotulinumtoxinA are limited.

Because most payers have minimum reinjection time limits for reimbursement, an understanding of the duration of effect of onabotulinumtoxinA in treating CD and the factors that may influence it, including dose, can provide clinicians and payers guidance for treatment and coverage decisions, respectively. Thus, we performed a systematic review and meta-analysis to evaluate the duration of clinical effect of onabotulinumtoxinA in patients with CD, and to identify potential factors that may influence it.

## Methods

### Literature search

Nine databases (MEDLINE®, EMBASE™, EMBAL, BIOSIS Previews/RN®, SCISEARCH®, PASCAL, International Pharmaceutical Abstracts [IPA], Dissertation Abstracts [DISSABS], and HCPlus®) were searched from January 1980 to January 2013 inclusive for published articles (prospective or retrospective studies) on the treatment of CD with onabotulinumtoxinA. Medical subject heading (MeSH) search terms were used to search for articles on CD: cervical dystonia, torticollis, laterocollis, retrocollis, anterocollis, spasmodic torticollis, wryneck. Search terms for onabotulinumtoxinA included: BOTOX®, botulinum, botulinum neurotoxin A, and onabotulinumtoxinA. The search results were combined and duplicate articles were removed. Included articles were peer-reviewed, non-review, English-language studies of the use of onabotulinumtoxinA for the treatment of CD published between January 1980 and January 2013.

### Study selection

**Figure 1:**
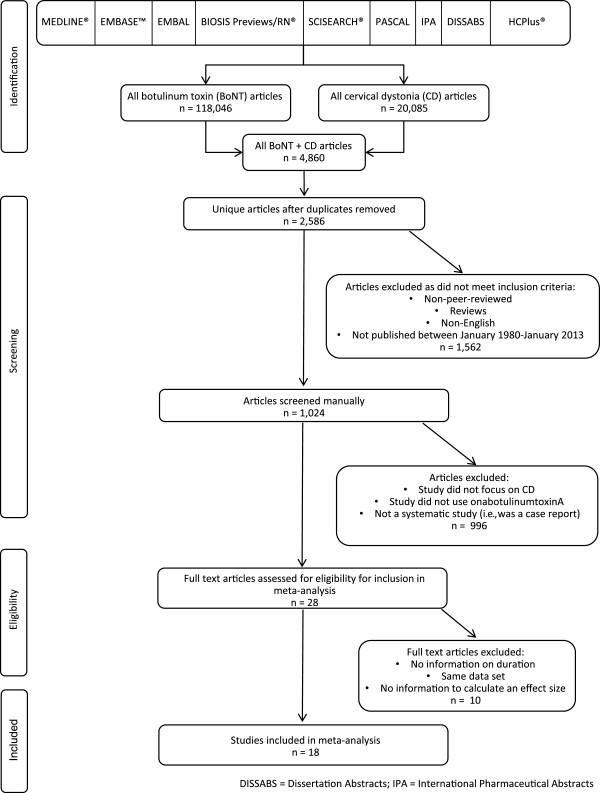
Flow diagram showing systematic search strategy and selection of articles.

Abstracts of all articles were downloaded and manually reviewed to eliminate articles that did not focus specifically on the treatment of CD (i.e., false positives) and did not include the use of onabotulinumtoxinA for treatment of CD; non-systematic studies (i.e., case reports) were also excluded. The full text of the remaining articles was then manually reviewed to determine if they included information on the duration of benefit of onabotulinumtoxinA in the treatment of CD (Figure 1), which was the focus of the analysis. The definition of duration of benefit was as provided by the authors within each individual article. Data on the duration of benefit of onabotulinumtoxinA, data needed to calculate an effect size (mean duration of effect and standard deviation of duration), the number of patients and dose of onabotulinumtoxinA used in each study, and the country where each study was performed were extracted from each published article by WM and LW. If any of the needed information was not included in the article, or if any additional data were needed for the meta-analysis, attempts were made to contact the corresponding authors.

### Assessment of data quality

To ascertain the validity of eligible randomized trials, pairs of reviewers working independently determined the adequacy of randomization and concealment of allocation; blinding of patients, health care providers, data collectors, and outcome assessors; and extent of loss to follow-up (i.e., proportion of patients in whom the investigators were not able to ascertain outcomes). To explore variability in study results (heterogeneity), we specified the following hypothesis before conducting the analysis: that effect size may differ according to the methodological quality of the studies.

The quality of each chosen article was evaluated using a modified version of the 24-item Cho & Bero instrument [10], which is an accepted method for evaluating article quality [11-16]. The bulk of the questions on the instrument are scored as ‘yes’, ‘partial’, or ‘no’ and have values of 2, 1, and 0, respectively; questions may also be scored as ‘not applicable’ to a particular article, which has a value of 0. The total score each article can receive ranges between 0 and 45. The two researchers then compared their scores for each article. Where scores differed by two points or less, an average of the two scores was used. Where scores differed by greater than two points, a discussion was held between the two researchers to assess questions in which the researchers had disagreements. Differences in scoring were resolved by consensus to produce an agreed-upon quality score.

### Meta-analytic methods

A formal meta-analysis to determine duration of effect of onabotulinumtoxinA was performed using Comprehensive Meta-Analysis version 2.0 (BioStat International, Inc., Tampa, FL, USA). A standard effect size based upon means and standard deviations was performed on the duration of treatment. Both fixed effects and random effects models were performed on the data. The Q-statistic for heterogeneity, I-squared, and Tau-squared were investigated to determine whether the overall effect size had significant heterogeneity across the fixed and random effects models. In the presence of significant heterogeneity, various article demographics and dosing of onabotulinumtoxinA were investigated through meta-regression and subgroup analysis for possible sources of variation among the published articles. Subgroup analyses were performed to identify variables that influenced the duration of effect of onabotulinumtoxinA.

### Assessment of potential publication bias

We performed several tests to assess for potential publication bias, including a classic fail-safe N (which is the number of additional studies that would be required to increase the p value for a meta-analysis to a level of non-significance [greater than 0.05]), the Begg and Mazumdar rank correlation, and funnel plots. A funnel plot was used to determine whether selective publication of small studies with positive results along with non-publication of small studies with negative results could have unduly influenced the outcome of the meta-analysis. Ideally, a funnel plot should show the studies equally distributed around the mean effect size and forming a funnel with the smaller sample size studies near the bottom due to their greater standard error.

## Results

### Literature search results

The initial literature search identified 2,586 unique articles on the treatment of CD with botulinum toxins (Figure 1). After review, 1,562 articles were discarded as they did not meet the inclusion criteria, and after examining the abstracts of the remaining 1,024 articles, a further 996 articles were excluded as they did not focus on CD, did not use onabotulinumtoxinA for the treatment of CD, or were not systematic studies (i.e., case reports), leaving 28 potentially-relevant articles. All 28 articles were then reviewed to see if they contained the information needed for the meta-analysis, including information on the duration of effect of onabotulinumtoxinA for the treatment of CD, along with information to calculate an effect size (mean duration of effect and standard deviation of duration). Four studies did not contain information on the duration of effect of onabotulinumtoxinA and were eliminated. Two articles reported data from the same set of patients as other articles and were eliminated to avoid duplication of data sets. Seven studies did not contain the necessary information to calculate an effect size for duration (e.g., means when medians were published, standard errors, etc.); between contacting authors and examining internal company study results, missing data for three of the studies were obtained. Thus, 18 unique studies met all inclusion criteria and had usable results (Table 1) [3,6-8,17-30]. From the 18 studies, 19 duration of effect estimates were obtained because one article contained two arms of a trial for which independent data could be extracted. Similarly, three of the 18 studies that contained duration information did not contain detailed information on the mean dose of onabotulinumtoxinA that was used [25,29], but the dose data were obtained from authors and/or internal company records.

**Table 1:** Studies included in meta-analysis

**First author, year**	**Study type**	**No. of patients**	**Mean onabotA dose/injection**	**Duration of effect**	**Definition of duration**	**Country of origin**
			**Mean (±SD) units**	**Mean (±SD)**		
Benecke 2005 [17]	Prospective, randomized, double-blind, parallel-group, noninferiority study	232	138.9 ± 46.8	94.3 ± 31.4 days	Interval between injection and first visit at which TWSTRS severity score reached 80% of baseline	Ex-US
Bihari 2005 [6]	Prospective, single-arm, crossover study	12^a^	131	64.3 days	Time to reach 70% TWSTRS score	Ex-US
Brashear 2005 [3]	Retrospective	172^b^	241.80-254.07	108.48 days (year 1)	Interval between treatments	US
Brashear 2000 [25]	Retrospective	60	263	15.5 ± 3.4 weeks	Interval between visits	US
Brashear 1998 [27]	Prospective, single-center study	149^c^	219.8 ± 63.5	142.9 ± 85.8 days	Average time between treatments	US
Brin 2008 [29]	Prospective, open-label, multicenter study	326	187.0 ± 76.5	110.2 ± 91.8 days	Time from treatment injection to date of next injection	US/Ex-US
Brockmann 2012 [24]	Retrospective	20	175 ± 76	10.7 ± 1.9 weeks	Time between last treatment and first subjective notice of reduction of benefit from the treatment	Ex-US
Camargo 2008 [18]	Prospective, single-center study	85	151.05 ± 52.55	89.1 ± 21.8 days	Patient-reported time to return of symptoms	Ex-US
Dubinsky 1991 [28]	Prospective cohort	84	269 ± 39	107 ± 49 days	Time from initial injection until patient-reported worsening of symptoms	US
Jankovic 2003 [19]	Prospective, single-center study	119	223.9 ± 73.0	14.6 ± 5.6 weeks	Not specified	US
Jankovic 1990 [26]	Prospective cohort	205	105.4 ± 33.8	14.0 ± 6.9 weeks	Duration of peak effect	US
Maia 2010 [20]	Retrospective	67	204.79	13.01 weeks	Duration which patient had any benefit from treatment	Ex-US
Meija 2005 [21]	Retrospective	16^d^	221.2 ± 129.4	15.4 ± 3.4 weeks	Not specified	US
Mohammadi 2009 [7]	Prospective cohort	44	145 ± 44	10 ± 2.4 weeks	Not specified	Ex-US
Naumann 2002 [8]	Prospective, randomized, double-blind, crossover study	59	155 ± 51	13.9 ± 2.6 weeks	Time to TWSTRS severity score ≥10 and rotational score ≥1	Ex-US
		68	157 ± 44	13.6 ± 2.4 weeks		
Odergren 1998 [22]	Prospective, randomized, double-blind, parallel-group study	35	152 ± 45	80.7 ± 14.4 days	Time to retreatment	Ex-US
Quagliato 2010 [30]	Prospective, randomized, double-blind study	12^e^	NR	11.7 ± 3.4 weeks	Interval between day of treatment and patient-reported waning effect	Ex-US
Ranoux 2002 [23]	Prospective, randomized, double-blind, crossover study	51	104.4 ± 20.6	89.1 ± 19 days	Interval between day of treatment and patient-reported waning of effect	Ex-US

^a^48 total patients; only 12 had CD. ^b^Case report forms. ^c^156 total patients; 149 had idiopathic CD. ^d^45 total patients; 16 had CD. ^e^24 total patients; 12 received onabotulinumtoxinA.

*Abbreviations*: *NR* not reported, *onabotA* onabotulinumtoxinA, *TWSTRS* Toronto Western Spasmodic Torticollis Rating Scale.

### Demographic and meta-analytic results

Publication dates for the 18 chosen studies were between 1990 and 2012. The quality scores for the 18 articles ranged from 11 to 42. Duration of effect for onabotulinumtoxinA across the articles ranged from 64.3 days to 142.9 days (9.2-20.4 weeks).

The fixed effects model produced a mean duration of effect of 93.2 days (95% CI 91.8-94.6 days), whereas the random effects model showed a mean duration of 95.2 days (95% CI 88.9-101.4 days). The Q-statistic, I-squared, and Tau-squared were investigated to determine whether the overall effect size had significant heterogeneity across the fixed and random effects models. The Q-statistic was 333.7 (p < 0.01), whereas the I-squared and Tau-squared were 94.6 and 176.2 respectively, indicating significant variation in duration of benefit for onabotulinumtoxinA between studies and the need to explore moderating variables.

**Figure 2:**
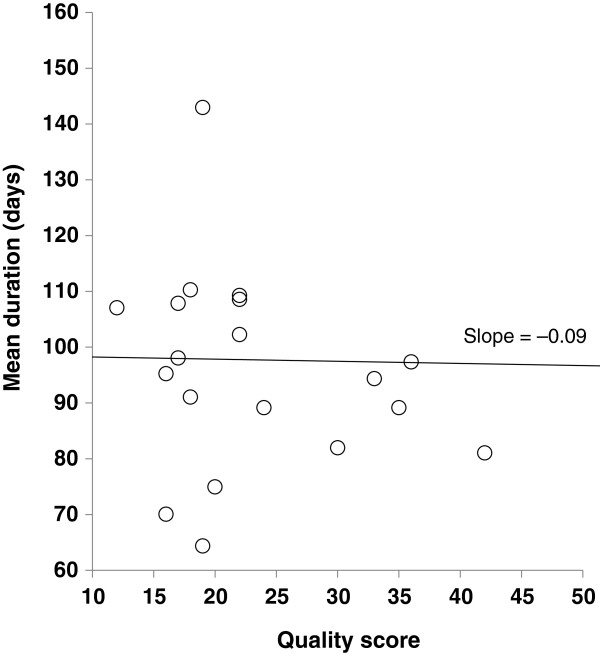
Regression of quality score on mean duration.

**Figure 3:**
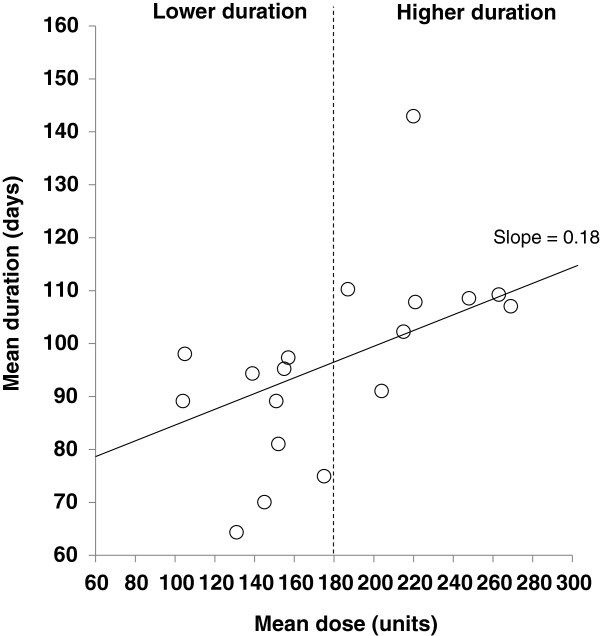
Regression of mean dose on mean duration.

Several demographic variables were chosen to determine whether they contributed to the significant variability across the studies. First, article quality score was subjected to a meta-regression. The slope of the regression line was –0.09, which was non-significantly different from zero (p = 0.25), indicating that quality was not a moderating variable (Figure 2). Second, the mean dose of onabotulinumtoxinA was subjected to a meta-regression (n = 18). The slope of the line was 0.18, which was significantly different from zero (p < 0.01) (Figure 3), indicating that dose was a significant moderating factor. Based on a visual inspection of the figure, there was a clear difference in mean duration of effect between studies using a mean dose <180 Units and those using a mean dose ≥180 Units, so a dose of 180 Units was chosen and articles were then dichotomized around this cut-off point. For studies using a mean dose <180 Units, the mean durations of effect for the fixed and random effect size models were found to be 87.8 days (12.5 weeks) and 85.7 days (12.2 weeks), respectively. For studies using a mean dose ≥180 Units, the mean durations of effect for the fixed and random effects models were 107.2 days (15.3 weeks) and 108.8 days (15.5 weeks), respectively. Subgroup analysis showed the duration of effect in these two groups to be statistically different (p < 0.01). Results of analyses using doses of 190 and 200 Units as the cut off were similar, with no change in statistical significance (duration was 88.4 and 87.7 days for the fixed and random effects models for studies using a mean dose <190 or <200 Units, and 106.9 and 108.8 days for the fixed and random effects models for studies using mean doses ≥190 or ≥200 Units).

Next, study type (experimental vs. an observational/retrospective database analysis) was then assessed to see if it had a moderating effect on duration. The fixed and random effects mean durations of effect were 92.1 days (13.2 weeks) and 89.5 days (12.8 weeks), respectively, for experimental studies. For observational and retrospective database studies, the fixed and random effects mean durations of effect were 94.2 days (13.5 weeks) and 99.3 days (14.2 weeks), respectively. These two groups were not statistically different (p = 0.14). Finally, country of origin of the study was investigated. Studies conducted in the US were compared with studies conducted elsewhere in the world. The fixed and random effects models showed mean durations of effect for US-based studies of 107.3 days (15.3 weeks) and 109.6 days (15.7 weeks), respectively, whereas non-US studies showed mean durations of effect of 87.2 days (12.5 weeks) and 84.8 days (12.1 weeks) for the fixed and random effects models, respectively. However, six of the seven studies with doses ≥180 Units were done in the US, and only one of the studies with doses <180 Units were performed in the US, which suggests the country-of-origin effect is explained by the doses administered.

**Figure 4:**
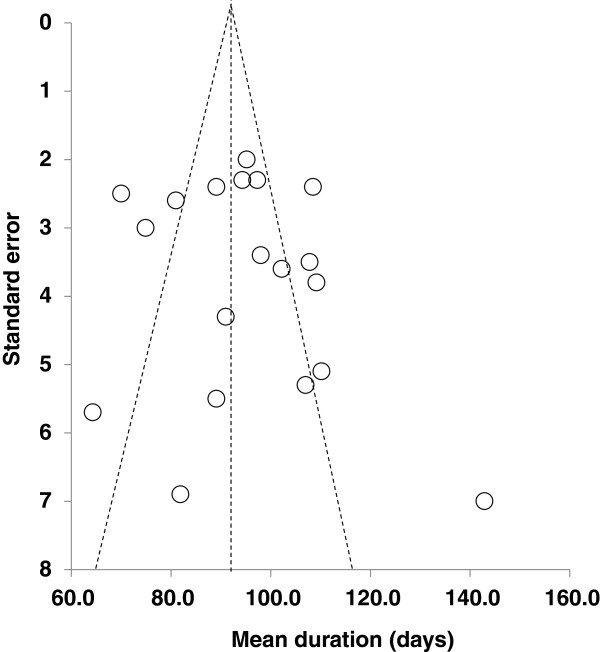
Funnel plot of standard error by mean duration.

Assessment of potential publication bias suggested no bias was present. The classic fail-safe N was 4,941 studies, and the Begg and Mazumdar rank correlation, which is interpreted similar to regular correlation coefficients, was non-significant with a p value of 0.73. The funnel plot showed that the data points were equally distributed around the funnel, but as many data points fall outside the funnel plot (Figure 4), there might not be one effect being measured. This is not surprising, however, given the substantial heterogeneity among studies and because several moderating variables were significant.

## Discussion

Botulinum toxins are considered first-line therapy for the treatment of CD [31] and must be injected on a repeat basis; therefore, duration of clinical benefit is a critically important factor for both physicians and their patients. Because there is heterogeneity in the published literature on how duration is reported, and because no prospective trials have been performed to assess the duration of benefit of onabotulinumtoxinA, we performed a systematic review and meta-analysis to identify a mean duration of clinical effect of onabotulinumtoxinA in patients with CD and potential factors that may influence it. None of the studies included in our analysis employed fixed injection intervals or a predefined study duration, thus enabling a naturalistic assessment of the waning of the clinical efficacy of onabotulinumtoxinA. In addition, as no dose-ranging studies have been performed with onabotulinumtoxinA, dose data were captured to allow assessment of the dose-duration relationship. The meta-analytic approach allowed us to weight studies appropriately based on their quality, design, and number of patients.

Botulinum toxins are a unique class of biologic agents in which a single injection cycle can provide several months of clinical improvement in symptoms in a variety of different disease states. Understanding the duration of clinical benefit of botulinum toxins in the treatment of CD and its impact on health care utilization are of importance in the contemporary health care environment. The required frequency of retreatment may influence treatment or product selection decisions and have practical implications for patients, physicians, and payers. This is even more relevant with the current emphasis in health care on incorporating patient-centered outcomes. A longer duration of effect will result in less frequent reinjection and should thus be associated with decreased annualized drug costs, decreased health care utilization and overall treatment costs, as well as improved patient satisfaction. This reduces the burden on the health care system, on payers and on patients by decreasing demands on physician time, which may improve access to care, lower administrative costs, and result in less inconvenience and less missed time from work for patients.

Our meta-analysis, which included 18 studies and over 1,900 patients, found that the mean duration of effect of onabotulinumtoxinA in the treatment of CD was 93-95 days (13.2-13.5 weeks). We also found that higher doses of onabotulinumtoxinA were associated with a significantly longer duration of effect. For doses ≥180 Units, the mean duration of effect was 15.3 weeks, while the mean duration of doses below 180 Units was 12.5 weeks. Dichotomizing by the 180 Unit dose was selected as practical after a visual inspection of the data distribution, as there is no statistical mechanism that could be used to more rigorously determine a dose to use for analysis. OnabotulinumtoxinA doses ≥180 Units may thus generally be preferred by patients, physicians, and payers, as this should reduce inconvenience for patients, increase patient satisfaction, and lower health resource costs due to longer duration of clinical benefit. It should be noted that performing an overall benefit-risk assessment as a function of dose, including an analysis of safety data, was not performed as it was not the objective of our analysis. However, our finding that doses ≥180 Units provide longer duration is also broadly consistent with the 236 Unit mean dose (range: 198-300 Units) reported in the CD pivotal phase 3 study of onabotulinumtoxinA in the treatment of CD, which was deemed to have an acceptable safety profile [4], and it should also be noted that patient dosing and injection pattern for CD is individualized on a per-patient basis.

In contrast, the published literature on other botulinum toxin products shows inconsistent dose-duration and dose-effect relationships in the treatment of CD. For example, a trial of incobotulinumtoxinA for the treatment of CD compared two doses (120 Units and 240 Units) versus placebo and found no advantage in objective efficacy or duration of effect with the higher dose [32]. A dose-ranging study of abobotulinumtoxinA (which examined the efficacy and safety of doses of 250, 500, and 1,000 Units) found no difference in efficacy between the 500 and 1,000 Unit doses, although both the 500 and 1,000 Unit doses provided better efficacy than the 250 Unit dose. The authors also observed that mean duration of effect appeared longer in the 1,000 Unit group, but none of the differences reached statistical significance; the study was also fairly short (only 8 weeks) and the measures of duration were all indirect [33]. Data from two long-term, multicenter, open-label extension studies of abobotulinumtoxinA for the treatment of CD, which allowed dose adjustment ranging from 250 to 1,000 Units following a fixed dose of 500 Units in the placebo-controlled trials, also failed to find a dose-duration effect [34].

Country of origin of the study was also found to affect the duration of effect of onabotulinumtoxinA. Specifically, the mean duration of effect was found to be longer for US-based studies than for studies performed outside the US (15.7 weeks vs. 12.5 weeks, respectively). This is not entirely surprising as physicians in clinical practice outside the US tend to use lower doses than those in the US, and our analysis revealed that higher doses are associated with longer duration of effect.

The four main limitations of meta-analysis are 1) combining studies that used different measuring techniques and/or definitions of variables; 2) combining studies of different designs and subjective quality; 3) publication bias; and 4) using studies that contain data on the same set of patients, which is commonly known as the non-independence of data problem. For this meta-analysis, all studies used individualized definitions of duration of effect, some of which were limited by the design of the studies themselves (i.e., prospective vs. retrospective). For example, in the prospective study by Naumann and colleagues, duration of effect was defined as the time from first injection to qualification for a second treatment (which was defined as a Toronto Western Spasmodic Torticollis Rating Scale severity score ≥10 and rotational score ≥1) [8], whereas in the retrospective study by Brashear *et al*., duration of effect was defined as the ‘interval between visits’ [3]. Several studies that cited a mean duration also failed to clearly specify an exact definition of ‘duration’ [7,20,21]. Differences such as these could have influenced the overall duration estimate. For the second limitation (combining studies of different designs and subjective quality), we found that the quality of each article did not have an effect on the overall effect size measured. The third potential limitation is publication bias. As described above, we performed several tests to assess for publication bias, including classic fail-safe N, the Begg and Mazumdar rank correlation, and funnel plots, all of which suggested that there was no bias. The final limitation of meta-analyses (non-independence of data) was handled by carefully inspecting article methodology and patient characteristics for ‘subject data duplication’. Two published studies that were identified as potential articles to add to the meta-analysis were eliminated from consideration due to apparent data duplication [35,36]. Additionally some large, well-controlled studies such as the ‘ABCD’ trial [37] were not included in the meta-analysis as they did not contain all of the necessary information for inclusion in the meta-analysis (i.e., included median instead of mean and/or did not include a measure to be able to calculate effect size), and we were unable to obtain these data from the investigators.

Notwithstanding the potential limitations, the robustness of this analysis is demonstrated by the strict meta-analytic technique and the large number of studies and patients included in the analysis. Furthermore, the potential limitations were systematically mitigated by robust statistical analyses as described above. Lastly, it should be noted that the results presented here are specific to onabotulinumtoxinA and cannot be extrapolated to any other botulinum toxin, as dosing units of onabotulinumtoxinA are not interchangeable with those of any other botulinum toxin preparation.

## Conclusions

This systematic review and meta-analysis demonstrated that the duration of effect of onabotulinumtoxinA in the treatment of CD is 93-95 days (13.2-13.5 weeks), with higher doses associated with a significantly longer duration of effect. This suggests that, in general, patients receiving treatment with onabotulinumtoxinA for CD would be anticipated to require approximately 4 treatments per year. This information may provide clinicians and payers guidance for treatment and treatment decisions with onabotulinumtoxinA.

## Abbreviations

CI: Confidence interval; CD: Cervical dystonia.

## Competing interests

This analysis was funded by Allergan, Inc. Wallace Marsh received travel support from Allergan, Inc. to present the preliminary meta-analysis results at an international conference. Conor Gallagher, Deirdre Monroe, and Mitchell Brin are employees of Allergan, Inc.

## Authors’ contributions

CG and DM participated in the design of the systematic review and analysis of articles. WM performed the meta-analysis. CG, DM, WM, and MB analyzed and interpreted the data, reviewed and edited the manuscript, and approved the final manuscript. All authors agree to be accountable for all aspects of the work.

## Pre-publication history

The pre-publication history for this paper can be accessed here:

http://www.biomedcentral.com/1471-2377/14/91/prepub
